# The bio-psycho-social determinants of health: Reflections on the CIAR Population Health Program (1987–2003)

**DOI:** 10.17269/s41997-025-01124-3

**Published:** 2025-10-15

**Authors:** John Frank

**Affiliations:** 1 Usher Institute, University of Edinburgh, Edinburgh, UK; 2 Dalla Lana School of Public Health, University of Toronto, Toronto, ON Canada

**Keywords:** Population health, (Social) Determinants of health, Canadian Institute for Advanced Research (CIFAR), Santé de la population, Déterminants sociaux de la santé, Institut canadien de recherches avancées (CIFAR)

## Abstract

In the mid-1980s, a remarkable polymath, Dr Fraser Mustard, founded the Canadian Institute for Advanced Research (now CIFAR – https://cifar.ca/), as a unique way to develop, across Canadian and selected international universities, over a dozen multi-disciplinary groups of researchers tackling major intellectual challenges of that era. Among these groups was the Population Health Program (PHP), led for its initial decade by the brilliant Canadian health economist Prof Bob Evans. Over the next decade-and-a-half, the PHP met nearly fifty times with top international scholars in all types of health research. Out of these interactions, the Program’s membership formulated a set of ideas about how the health of entire human populations and societies is determined, as well as enunciated clear policy and program implications of those ideas. This paper summarizes, from the point of view of the author (a Scholar, then Fellow in the PHP) the main themes that the PHP enunciated during its initial decade, culminating in the widely read volume “*Why are Some People Healthy and Others Not”?. Finally, developments in the field of Population Health in the two decades since the “sunsetting” of the PHP are reviewed and commented upon.*

## Résumé

Au milieu des années 1980, un polymathe remarquable, le Dr Fraser Mustard, fondait l’Institut canadien de recherches avancées (aujourd’hui le CIFAR – https://cifar.ca/), un établissement unique en son genre ayant pour mandat de mettre en place dans les universités canadiennes et dans certaines universités étrangères plus d’une douzaine de groupes pluridisciplinaires de chercheurs qui s’attaquaient aux grands défis intellectuels de l’époque. L’un de ces groupes fut le Population Health Program (PHP), un programme de santé des populations dirigé pendant ses 10 premières années par un brillant économiste canadien de la santé, le professeur Bob Evans. Au cours des 15 années suivantes, le PHP a tenu près de 50 réunions avec des spécialistes internationaux de premier plan faisant de la recherche en santé. À partir de ces échanges, les membres du PHP ont formulé un ensemble d’idées sur les facteurs qui déterminent la santé de populations et de sociétés humaines entières, puis énoncé les implications claires de ces idées pour les politiques et les programmes. Sont résumés dans cet article, du point de vue de son auteur (théoricien, puis membre du PHP) les grands thèmes énoncés par le PHP au cours de ses 10 premières années d’existence, lesquels ont abouti à un ouvrage très connu: *Why are Some People Healthy and Others Not?*. Enfin, l’auteur passe en revue et commente l’évolution du domaine de la santé des populations dans les 20 années qui ont suivi la disparition du PHP.

## Introduction

When Fraser Mustard began his search for Canadian (and some global) scholars to form the initial PHP, it is noteworthy that he began with a health economist (Bob Evans of the University of British Columbia), quickly followed by a political scientist who was expert in policy analysis (Prof Ted Marmor of Yale University). It was only later that the group recruited members from academic disciplines – such as epidemiologists, geneticists, sociologists, anthropologists, and health services/policy researchers – more traditionally engaged in investigating the “Determinants of Health” (a phrase first used by Mustard and the author, a medical epidemiologist, in their original mimeographed document, published by CIAR in 1991, laying out the main ideas of the Program. [See: Mustard JF and Frank JW (on behalf of the Population Health Program, Canadian Institute for Advanced Research.) *The Determinants of Health*. (Working Paper) CIAR, Toronto, 1991. Many of these ideas were later published in a peer-reviewed journal (Frank, 1995)].

Mustard’s reasoning, as a widely published pathologist, was that most of what traditional biomedical researchers had discovered about the “determinants” of health was actually focused on the *mechanisms* (molecular, sub-cellular, cellular, and physiological/biochemical) by which various external exposures and experiences, interacting with our genetic (and, in retrospect, epi-genetic—although the term was not yet in wide use) legacies, determine the diseases and conditions that define our health and function, over the life-course. Mustard wanted the PHP to pursue a new approach to this question, one that was much less focused on pathogenetic mechanisms, and more oriented to characterizing the underlying drivers – exposures and experiences – that determine human health, to inform policies to improve health and reduce health inequalities. Fundamental to that effort was the exploration of “heterogeneities in health status” as laid out in Chapter 3 of the PHP’s first book, “Why are Some People Healthy and Others Not?” (Hertzman et al., 1994).

This paper attempts to document the key concepts which the PHP developed over several years of regular discussions with diverse health scholars during twice-yearly Program meetings. Those meetings were often rather rambunctious, since the model of intellectual discourse in which the Program Leader, Bob Evans, had been trained was the “bear-pit” approach, emphasizing a guest speaker’s ability to field often-abrupt questions and comments from the audience (typically about twenty Program members), who were encouraged to interrupt guest speakers’ formal presentations at any point! Tracing the precise origins of the key Program ideas that emerged during that first decade is therefore rather fraught – often they were the product of group interactions, not well documented in any sort of “meeting minutes.” For that vagueness of attribution, the author apologizes in advance, in that – even though he attended virtually every one of those PHP meetings for nearly fifteen years as a Scholar then Fellow of the Program, memory is not the finest basis for scholarly documentation.

## Four main classes of determinants of health

PHP members gradually promulgated four main types of “Determinants” of human health, especially those that produce systematic differences in health status across entire populations:

I. The limited role of medical care as a determinant of health.
II. The powerful role of societal/cultural characteristics.
III. The key role of individual socio-economic status and the immediate social environment of the person.
IV. The influence of daily stressors and supports over the life-course (embedded early in life in our biology via psycho-neuro- endocrinological/immunological pathways).

Each of these four classes of key determinants of health is reviewed below, from two complementary perspectives: that of the PHP itself during its first decade of meetings; and that of the current scientific knowledge base underpinning population health. The intent is to reflect on how these core PHP ideas have fared, during the three decades since they were first enunciated in the collected writings of Program members.

## The limited role of medical care as a determinant of health

The historical origins of this concept date back to the writings of Thomas McKeown, a Canadian epidemiologist working in the UK and writing mostly in the 1970s (McKeown, 1975, 1979) well as a globally influential Canadian federal policy document published in 1974, attributed to the Minister of Health at the time, Marc Lalonde (but now generally credited to senior policy analysts working with Lalonde, such as Irving Rootman) (Government of Canada, 1974). This landmark document was, as the eminent Canadian public health thinker Trevor Hancock put it (Hancock, 1986):

“…the first significant government report to suggest that health care services were not the most important determinant of health. After reviewing the evidence, the report suggested that there were four “health fields”–lifestyle, environment, health care organization, human biology—and that major improvements in health would result primarily from improvements in lifestyle, environment and our knowledge of human biology.”

While these four “health fields” were not adopted by the PHP as their own “key determinants of health,” the Program’s thinking was influenced by Lalonde’s and McKeown’s rejection of health *care* as one of these determinants. In McKeown’s case, this was largely based on his in-depth analysis of the historical decline in mortality, as extensively documented in the UK after the Registrar General began complete registration of deaths and death-rate computation based on the census, in the 1830s. Writing about this groundbreaking work by McKeown three decades afterwards, Frank and Ostry (2010) credit McKeown with being essentially correct in his assessment of the limited role of medical care – as practiced at that time – in the major decline in overall population (and particularly infant and toddler) mortality, at least as documented in the UK. However, as the title of their article points out (“Was McKeown Right for the Wrong Reasons?”) many medical historians would now attribute that decline to major reductions in family size, and – critically – child spacing perhaps motivated by economic recession, achieved by the British between about 1880 and 1930, despite the limited availability of any effective birth control (Frank & Ostry, 2010). In short, therefore, McKeown’s work probably has rather little to say about the role of modern medical care as a determinant of the overall health status of entire populations in the “developed world”. [On the other hand, child spacing has become, over the intervening half-century, a well-evidenced basis for achieving reductions in infant and child mortality in lower-income countries (Frank & Ostry, 2010)].

In their own development of the key determinants of health, listed above, PHP members found the four put forward by Lalonde to be unconvincing, apart from the limited role of medical care. In particular, they objected to:

- The separation by Lalonde of “lifestyle” and “environmental” determinants of health, in that – as modern social scientists now agree – decisions people make about their diet, smoking, alcohol and drug use, sexual practices, etc. are not purely of their own free will or choice, but rather are extensively influenced by often commercially motivated manufacturers, distributors and advertisers of these unhealthy products and services, now termed “marketable hazards” or “the commercial determinants of health” (Moodie et al., 2013). The PHP did not want to be identified with “victim blamers” in this regard.
- The vagueness of Lalonde’s final “health field” (class of determinant): “human biology” – PHP members, several of whom had advanced training in human biology, felt this term was so general as to be completely unhelpful, as it indiscriminately embraces all aspects of the causation of ill health, from genetics through to toxic effects of physical and social environments on human functioning.
- The failure of Lalonde to specify which aspects of the “environment” are particularly important determinants of health – for example, PHP members with economic training were already convinced that more redistributive taxation and social welfare policies were absolutely central to achieving improvements in population health status overall, and especially the reduction in socio-economic inequalities in health (about which more later), as clearly demonstrated in several chapters of the PHP’s second book, published in 2006 (Heymann et al., 2006).

In fact, PHP writings steadfastly refused to cite any of the several “pie-charts” published in the intervening years (McGinnis & Foege, 1993; McGinnis et al., 2002), allegedly depicting the proportion of “ill health” attributable to such broad classes of determinants. A widely cited example of such a pie-chart is described by McGinnis et al. (2002) thus:

“On a population basis, using the best available estimates, the impacts of various domains on early deaths in the United States distribute roughly as follows: genetic predispositions, about 30 percent; social circumstances, 15 percent; environmental exposures, 5 percent; behavioural patterns, 40 percent; and shortfalls in medical care, 10 percent.”

The PHP found such analyses to be scientifically dubious. In fact, the many published versions of them are in profound disagreement. For example, such “pie-charts” often do not define the precise health outcome being partitioned as to the contributions of various “determinants” to its overall population burden of illness/death – is it overall mortality, premature mortality, quality-adjusted life-years lost, or what? Indeed, in a meeting late in the PHP’s history, members were regaled with a paper published by Canadian public health authorities, suggesting that the PHP itself had authored such a “pie-chart” – which was completely untrue. As the members of the Program were well aware, any attempt to produce such a pie-chart with robust epidemiological methods would soon run aground, due to both longstanding methodological conundrums (e.g. “Population Attributable Risk” calculations are notoriously subject to multiple underlying assumptions, often impossible to test (Northridge, 1995)) as well as differences in how various sorts of determinants influence overall population health over time, and across settings. For example, those of us who remember the early years of the HIV/AIDS pandemic, before anti-retroviral drug therapy was invented and widely distributed globally, can testify to the overwhelming influence of sexual and intravenous-drug-use behaviours as the key determinants of that pandemic’s massive new burden of illness, in both high-risk populations in rich countries, and then, by the 1990 s, hundreds of millions of citizens of the poorest countries. But, by the end of that decade, the key determinant of the health of HIV-infected persons worldwide became, instead, access to life-saving anti-retroviral therapy drugs. In short, generalizing globally, over different eras, about the “relative contributions” to overall health and wellbeing, of various fundamental drivers of health status, is virtually impossible – the “devil is in the details.”

What then is the current view of most population health scientists concerning the relative importance, as an overall determinant of health, of health care (and the care system attributes that determine access and use of care, such as point-of-care user fees, versus various health insurance models)? This writer would argue that the evidentiary ground on which one could reasonably base one’s answer to this question has only been augmented in a rather limited way, in the three decades since the PHP produced its first and arguably more influential book. A big part of the challenge to producing such evidence is that one cannot randomize entire populations to receive more versus less modern medical care. Rather, entire societies are affected, within any given period of time, by particular health care policies surrounding health care access and use – such as what the WHO has recently come to champion as “Universal Health Coverage” (WHO, 2015), which is also a key element within Sustainable Development Goal #3. The health status consequences of such naturally occurring variations in UHC provision and uptake must therefore be studied using “natural experimental” methods – most of which have been developed by econometricians, and many of which also have important underlying assumptions which are not easily tested. Those readers with an appetite for such complex studies are directed to an influential analysis of them (Lantz et al., 2007), as they are beyond the scope of this paper.

However, to close our discussion of this first “plank” in the PHP’s four-plank set of statements (listed above), which argues that health care is not a powerful determinant of health at the population level, there is one peer-reviewed study, published in Canada in 2007, which is strongly suggestive (if not entirely conclusive) that that view – that truly “modern” medical care is not an important determinant of health – may simply be wrong. James et al. (2007) conducted a robust analysis of the gap in age-standardized “years of life lost” (a measure of premature mortality) between the top and bottom quintiles of Canadian household income, aggregated to the neighbourhood level, between 1971 (shortly after UHC was truly established in Canada for “medically necessary” ambulatory and hospital care) and 1996. To isolate the effects of medical care from other influences on this health (mortality) disparity, James et al. separated all deaths over the 25 years into four categories, using a methodology utilized in many previous studies: deaths amenable to prevention by modern medical care (under most circumstances, albeit not in every case); deaths amenable to effective public health measures to change people’s exposure to health hazards (e.g. implementation and enforcement of strong evidence-based tobacco control strategies); ischemic heart disease (still the commonest single cause of premature mortality in most High-Income Countries, despite a near-universal fall of over 50% in its mortality rate over the last five decades, well characterized in multiple studies to be about half due to better medical care for individual patients, and about half due to reduced exposure to risk factors – such as smoking – caused by both public health measures and profound cultural change over time (Ford & Capewell, 2011). James et al. (2007) found that only the first class of deaths – those amenable to medical care – showed major steady declines in Canada, in 1971–96, just after Medicare was fully established. Of course, this does not prove that the UHC-like provision of “free” medical care in Canada over that time was responsible for those declines, which were similar in adults of both sexes. Indeed, the authors present no counterfactual evidence, such as an international comparison – e.g. with the USA, which has had no such UHC policies, even now – indeed, at least one recent study shows clearly that most inequalities in USA mortality have steadily increased over this same period (Singh & Siahpush, 2002). Neither is there a published “before/after” analysis featuring comparable Canadian death data from before and after the pre-Medicare era. However, most experts would not be able to come with a credible competing hypothesis to explain these findings, so that James et al. (2007) can be considered one of the few studies to date supporting Canada’ Medicare system’s benefits (and therefore the role of modern medical care) in reducing health inequalities.

In retrospect, it is tempting to speculate what background factors facilitated the emergence of the PHP’s thinking in the 1980 s, and specifically, what was it in the intellectual ambience of that time, in Canada (especially the ambience around Fraser Mustard) which “made the PHP happen.” This writer would point to Lalonde’s arguably revolutionary policy document in 1974 (Government of Canada, 1974; Hancock, 1986) as a critical inflection point in health policy that “permitted” even hardened biomedical researchers such as Mustard to seriously consider the key role of non-health-care factors in the determination of human health. Perhaps as well, Mustard’s twelve years helping to found McMaster University’ radical new medical school contributed to his commitment to training more critically oriented (and trans-disciplinary) health professionals for the modern era. Whatever the precise drivers of Mustard’s radical vision for Population Health, those of us who knew him well during this period will remember that he was “in full flight” from traditional university approaches to solving important but thorny research problems. Indeed, all the dozen programs of work at CIAR that he founded, ranging from Cosmology to Superconductivity, benefited from his firm view that dyed-in-the-wool university departments were inherently not supportive of, nor well-suited to, trans-disciplinary work that challenges normative thinking.

## The powerful role of societal/cultural characteristics (at the level of whole societies)

In the early PHP meetings, two important national examples were explored with visiting experts, of the effect of “historical societal trajectories” on entire populations’ health status: Japan’s remarkable increase in life expectancy from the 1960 s onward; and the former Soviet Union’s massive economic and social collapse of 1991–2, in which virtually every adult cause of death (apart from cancers, long known to have remarkably slow “response times” to changing social conditions, due to their long latency) increased suddenly, albeit concentrated in middle-aged men. The former case study has been expertly analyzed by Hasegawa (2001), a contributor to a major WHO publication on health inequalities, edited by Tim Evans et al. She convincingly makes the case that the strongest correlate of Japan’s remarkable rise in longevity is the sudden increase in universal primary (and, soon after, secondary) education among Japanese women and girls, deliberately achieved by the almost feudal government between 1890 and 1910, in order to accelerate national industrialization. This argument is in line with other convergent evidence that nationwide improvements in female education lead to longer intervals between births, which lead in turn to much reduced infant and toddler mortality (Adhikari and Sewangdee, 2011). [Further backing up Hasegawa’s hypothesis as to the cause of declining Japanese mortality in all adult age groups after the 1960s, there is also evidence, astonishingly, that survivors of birth cohorts with lower infant mortality live substantially longer lives, presumably through the biologically embedded effects of more salubrious environments in their early life (Meza et al., 2010)].

The Russian case study has been extensively analysed by demographers and sociologists, with the current consensus being that this massive and sudden collapse of that huge national economy, together with the huge political changes of that time as the old Soviet Union splintered into many newly independent countries, combined to create enormous “psycho-social stressors” that then manifested as higher rates of disease and death from almost all causes. Somewhat obfuscating the cause of that sudden major epidemic of premature mortality in Russia after 1991 is clear evidence that it was strongly mediated by the increased consumption of largely illegally produced alcohol, particularly via harmful binge-drinking (Leon et al., 1997). This interpretation has been substantiated by subsequent work on Russia’s demographic “recovery,” as it returned to much lower mortality rates after 2004 (Shkolnikov et al., 2013). Many other studies have confirmed the massive adverse health effects of economic recessions and other societal “shocks” that impact on large swathes of the population (Stuckler et al., 2009). A particularly intriguing econometric analysis (Bhattacharya et al., 2013) suggests a more subtle driver of the excess alcohol-mediated mortality in the USSR in the early 1990s: the termination of the remarkably effective anti-alcohol campaign by the Gorbachev government, over the previous half-decade, leading to a pent-up demand and massive binge drinking just as the USSR’s economy went into a tailspin. [This writer judges that analysis to be suggestive but not conclusive – it is likely that both factors contributed to the short-lived epidemic of alcohol-related mortality in 1991–4].

Whatever the proximal causal pathway of “auto-destructive behaviours” during such societal crashes, the fact remains that the fundamental driver of the 1990 s mortality crisis in the former USSR (and many of its client states) was a massive loss of income, financial and social security, and indeed, morale. Notably, this was the conclusion of a comprehensive review of declining health in all of Eastern Europe, by PHP Associate Director Clyde Hertzman (Hertzman, 1995).

These examples teach us that individual health-related behaviours – for example related to “lifestyle choices” such as tobacco use, poor-quality diet, etc. – are deeply contextualized within a society’s norms, leading to potentially dramatic changes in health status when these norms are suddenly shifted – for example, by massive reductions in income and social sense of security of the population. In short, even the “best-behaved citizens” in such situations can suffer health consequences because the “ship of life” (i.e. the society in which they live) on which they are making their life-journey has run aground – individual behaviours matter, but they are not simply a matter of the person’s free will and “choice.” More recent analyses of different EU nations’ economic policy responses to the 2008 recession suggest that such exogenous shocks to a society can be substantially mitigated, in their adverse health and other effects on the poor and marginalized, by the adoption of progressive taxation and social welfare programs (Karanikolos et al., 2013). In short, while recessions are bad, their health and social effects can be reduced and made more equitable by well-chosen public sector policy responses – these effects are not just an inevitable consequence of the business cycle.

Perhaps the most contentious post-PHP development, in terms of scientific evidence on this class of health determinants, is the “Spirit Level hypothesis” – so-called after the influential book by that name, published in 2010 by sociologist Richard Wilkinson and epidemiologist Kate Pickett (Wilkinson & Pickett, 2010). Briefly put, this hypothesis posits that any sort of inequality, in any society, tends to reduce the overall health, productivity, creativity, and quality of life of that society – more or less independent of the societal level of the determinants of health and function in and of itself. Those wanting a more scholarly treatment of this same topic should read the same authors’ 2015 “causal review” of the mountain of relevant evidence (Pickett & Wilkinson, 2015).

On the other hand, there are high-quality studies suggesting that this relationship is not quite universal internationally and over time, in that there may be “threshold effects,” [Personal Communication, Ichiro Kawachi, 2021] whereby only societies with relatively high levels of inequality tend to show strong relationships between more geographically localized measures of population inequality (for example, household income) and health outcomes (such as adult mortality from all causes.) This was precisely the main finding of an influential analysis of metropolitan-area (major cities) income inequality and adult mortality, published in 2005 (Ross et al., 2005): only the US and the UK showed any correlation at all. Australia, Canada and Sweden (all countries with significantly lower levels of income inequality) did not show a correlation. Therefore, while international scholarly support for the Spirit Level hypothesis has been increasing steadily over the last decade, there are still some experts who are unconvinced.

On a more personal level, in a recent High Court of British Columbia decision on the case against Cambie Surgeries (a provider of private surgical services, almost entirely joint replacements and cataract extractions, for those able to pay from their own pocket), the Spirit Level hypothesis was held up as a scientifically credible reason – based in part on the author’s expert testimony (see quotation below) – to maintain strong legal sanctions against for-profit, privately provided, essential health care in Canada. Cambie took the BC Government to court, alleging that the BC legislation (and the closely related Canada Health Act (1985), forbidding such for-profit provision of medically necessary care – while the physician also “extra-bills” the publicly administered “Medicare” system for the currently contracted rate – was counter to the Canadian Bill of Human Rights, because it abrogates the “right of (wealthy) Canadians to pay for earlier care.” Justice Steeves, in his verdict rendered in September 2020, found that:

“[2655] Overall, I find that there is evidence to suggest that duplicative private healthcare would exacerbate wealth and health inequality. I also accept the evidence of Dr. J. Frank that socioeconomic status is a significant determinant of overall health and well-being, and poor health status disproportionately affects lower income individuals. Further, duplicative private healthcare and the creation of a two-tier system, where access to preferential treatment would be based on the ability to pay, would exacerbate health inequity in terms of access to healthcare, utilization of healthcare and health outcomes.” (Cambie Surgeries Corporation 2020)

## The key role of individual socio-economic status and the immediate social environment of the person

This theme of the PHP was probably the most written-about of the four, by both Program members as well as independent scholars, including the extensive work of Sir Michael Marmot, an early Program member. Indeed, it was to an early meeting of PHP members (in the mid-1980s) that Marmot first presented (outside of the UK) his ground-breaking work depicting long-term, stable and epidemiologically large inequalities in diverse health outcomes, within the comparatively well-paid workforce of the UK Civil Service based at Whitehall (Marmot et al., 1984). The group’s reaction was electric – even the sympathetic epidemiologists in the group (including the author) expressed surprise that such major health inequalities could exist within a stable and relatively well-paid workforce that, although clearly hierarchical in nature, included no truly poor or unemployed persons. Over the following half-decade, however, as Program members began to look more systematically for evidence of such “socio-economic gradients” in health, it became apparent that these have been demonstrated in virtually every population/setting/time-period where they have been systematically investigated (Frank & Mustard, 1994; Hertzman et al., 1994).

Over the last three decades, many hundreds of social epidemiological studies have established that the following characteristics are almost always demonstrated for socioeconomic gradients in health, spanning an amazing range of populations, time-periods, disease outcomes, and measures of both health and socio-economic status (Frank, 1995; Frank & Mustard, 1994; Hertzman et al., 1994):

- “Fractal” tendencies revealing the same monotonic pattern (slope-sign) of risk, by SES level, no matter how many quantiles of rank-ordered SES are used in the analysis;
- Protean (pan-pathogenetic) – i.e. biologically non-specific – manifestations, consistent in the directionality of associations (the more deprived the individual, the higher the risk of the adverse health outcome) across many diverse diseases;
- Remarkable resistance to change in magnitude over time, even though predominant, specific diseases and traumas that make up such gradients may change a great deal.

In search of better understanding of these issues, the author was fortunate to be recruited in 2008 to Edinburgh, Scotland, to head up a new Centre/Unit, funded by the UK Medical Research Council and the Scottish Chief Scientist Office: the Scottish Collaboration for Public Health Research and Policy. Over the next ten years, until its core funding came to a preplanned end, the author was privileged to work with many diverse researchers and stakeholders in Scottish and UK public health research and policy, designing and conducting applied research to inform Scottish policies to improve health equitably, while also training over a dozen young scholars at the post-doctoral level to do this kind of research, and translate it into policy and practice (Frank, 2009; Frost et al., 2012).

A major lesson that Scotland taught me and my post-doctoral fellows over this period was that policy action to reduce socio-economic inequalities, and their health consequences, cannot be expected unless scientifically robust and cost-effective surveillance systems, measuring those inequalities and monitoring their time-trends, are solidly in place. “What does not get measured does not get attention.” As it happens, Scotland was the first nation globally to establish such a surveillance system (Frank & Haw, 2011), and it remains the only nation-state in the OECD which has accumulated multiple years of annual, methodologically-sound analyses to precisely document the extent to which these health inequalities have changed – or not (Frank & Matsunaga, 2022).

That is the good news from Scotland – it is entirely possible to set up an accurate and comprehensive surveillance system for health inequalities, based entirely on routinely collected data, suitably anonymized with modern record-linkage methods, at very low ongoing/maintenance cost. The less good news is that doing so is no guarantee that reductions in these health inequalities will result, even when – as in Scotland – there have been literally hundreds of public and voluntary sector policy documents published in support of the reduction in these inequalities, over the quarter century since Scotland’s devolution from the UK began (McCartney et al., 2011). On the other hand, analyses of how other EU nations have fared, in terms of tackling their own health inequalities, clearly show that hardly any society has had much success in this policy arena – the global structural forces that maintain the underlying steep socio-economic inequalities in income, education, occupational class, and levels of household deprivation simply appear to be too powerful for most western democracies to counter them – or at least the vested interests in those democracies are too powerful to enable governments to succeed in this respect (Mackenbach, 2012).

To be completely fair to the Scottish Government, until the last few years, it did not hold the key public policy levers which most experts believe represent the most important influences on such societal patterns of inequalities: taxes and social welfare (transfers). While those responsibilities are now finally being handed over to the Scottish Parliament from Whitehall, many fear the prognosis for strong Scottish Government action on inequalities in the near future is guarded, given the recent “perfect storm” of the COVID-19 pandemic, with its catastrophic effect on the UK national economy; the unresolved and deeply divisive Scottish independence struggle; BREXIT; and the unresolved legacy of the 2008 recession (especially in Scotland, where many decades of de-industrialization, followed more recently by the collapse of North Sea oil and gas revenues, that neither of these two previous “engines of the Scottish economy” can bring back the relatively well-off Scotland of yesteryear) (Frank, 2009; Keating, 2018).

## The influence of daily stressors and supports over the life-course (embedded early in life in our biology via psycho-neuro- endocrinological/immunological pathways)

This account of the key ideas of the PHP would be remiss if it did reflect on perhaps the most policy-actionable advice on which the Program converged. I am of course referring to practical policy guidance to democratic governments everywhere, about the most efficient investments for reducing health inequalities, and the underlying socio-economic inequalities that drive them, in the long run: *investments in high-quality, universally accessible, and well administered early childhood development and education programs*. (Blair et al., 2018; Frank et al., 2015; Hertzman et al., 2010; Keating & Hertzman, 1999) Long championed by Fraser Mustard himself (who spent his last decade working tirelessly for this cause, after leaving CIAR to set up the Founder’s Network (McCain & Mustard, 1999), this also became the life’s work of Prof Clyde Hertzman, the brilliant medical epidemiologist at the University of British Columbia (and Associate Director of the CIAR PHP) who set up the Human Early Learning Partnership Program (HELP) in Vancouver, nearly 20 years ago (Irwin et al., 2007).

Critical to this effort was the groundbreaking creation of the Early Development Instrument (EDI) by Prof Dan Offord of McMaster University (Janus & Offord, 2007): a generic, low-cost and low-tech tool for capturing and standardizing Grade 1 teachers’ holistic assessments of their classroom pupils’ levels of development across five domains which co-determine a child’s “readiness to learn” as they start school. Validated in many countries, and since the early 2000 s, the subject of a massive, multi-year research and evaluation program across Australia, the EDI provides a low-cost way to monitor the developmental status of each birth cohort of 5-to-6-year-olds entering school, while also providing community-specific feedback on precisely which domains of child development should be the focus of improved local pre-school programming, to improve outcomes for future birth cohorts (Collier et al., 2020). Sadly, Scotland did not embrace the use of the EDI despite a very successful pilot run by the author and his colleagues (Woolfson et al., 2013), largely because of a Ministerial commitment to the alternative view of how to help young children improve their school performance: test their “three Rs” (reading, (w)riting, and ‘rithmetic) skills on school entry, and focus their primary school curriculum on perfecting precisely those skills, beginning in Primary 1 (age 5–6.) This longstanding commitment of the Scottish education system has unfortunately persisted to the present day, despite plenty of international evidence that this age is too young for many children – particularly those from deprived backgrounds – to successfully master the three Rs, leading to student demoralization, inferior later educational performance, poor labour market options, and consequent lifelong disadvantage. () Consequently, this approach – focusing on the three Rs from age 4 to 5 onward – is not supported by many mainstream authorities in early childhood, especially those experts familiar with the Nordic countries’ tradition of delaying such formal education until about age 7 – countries with some of the highest overall educational attainment test results in the world, and certainly the most egalitarian test scores, across measures of parental social class (Frank & Geddes, 2020; Willms, 2003). Whether the current Scottish approach can improve the longstanding (and largely unchanging) steep gradient of national standardized test results by parental education level – first documented in Scotland over twenty years ago by Canadian Douglas Willms, and so clearly documented over recent decades by the OECD-run Program for International Student Assessment (PISA) – is still unclear (Willms, 2003).

## Recent developments in population health intervention research

Fortunately for the future of Population Health thinking, much recent work has gone into formulating a clear conceptual framework for implementing and evaluating policy and program *interventions* with the potential to both improve a whole society’s average level of health and reduce health inequalities by social class and other markers of relative powerlessness and/or relative deprivation. A landmark conceptual paper published in 2009 by Hawe and Potvin (Hawe & Potvin, 2009) was a clear advance in defining and explaining the importance of such research. More recent papers offer evidence that: the relative cost-effectiveness of such interventions can be very competitive with the return on investment from other health-sector policies (Masters et al., 2017). However, there remain major practical challenges of both doing conclusive research of this kind, and of synthesizing it so it can be used by decision-makers in the “real world.” (Ogilvie et al., 2020). Steady progress in this field is now encouragingly evident, despite the rather long lag between the original CIAR PHP writings, and these more recent developments. One clear and very positive consequence of the effort to champion Population Health intervention research (PHIR) is the remarkable increase in the number and value of PHIR grants awarded by the Canadian national health research funding agency, CIHR, over the first decade-and-a half after its reorganization in 1990, which included the founding of the first national funding body for research in population health: The CIHR Institute of Population and Public Health (Di Ruggiero et al., 2017). While there is still much more capacity development to do in this nascent field, there are strong signs that a productive base of researchers across Canada are now engaged in high-quality population health intervention research.

## Dissenting voices

It would wrong not to include in this overview reference to articulate and scholarly dissenters from the largely positive view of Population Health presented thus far. A useful and dispassionately neutral account is provided by Lucyk (2018) of the many such writings before and during the two decades since the PH stopped meeting, based on diverse key stakeholder interviews and archival reviews. Lucyk shows clearly that there was substantial resistance to, and criticism of, the CIAR PHP and its ideas, from the early 1990s onward – most of it, rather ironically, from Canadian social science academics. Those critics have been particularly concerned by: the relative dearth of qualitative-methods evidence in PHP writings, which tend to focus instead on largely quantitative, “positivist” science; the PHP’S apparent lack of a clear feminist perspective; its lack of attention to the neocolonial reality of global aboriginal peoples’ lives, beset by strong negative legacies of the earlier colonial conquest; its shying away from direct criticism (or indeed, even mention) of the capitalist system, and – sadly, since there was no truth in this – the allegation that CIAR’s programs depended on massive financial support from the private sector (in fact, CIAR’s major donors were always various provincial and federal governments.) A full discussion of these writings is beyond the scope of this paper, but those wanting more detail should refer to Lucyk and also an earlier, exhaustive review of the entire field and its critics (Hayes & Dunn, 1998).

## Conclusion

One can see from the above account of the key ideas in the PHP’s initial formulation of the determinants of health, and how these ideas have fared since then, that there are only some modest victories to declare—-and those only rather tentatively:

- The PHP’s view that medical care is not a major determinant of health is still influential, but has a rather dated ring to it, in an era of Universal Health Coverage, and ever-more-effective medical technology.
- The Program’s emphasis on such societal-level characteristics as the level of socioeconomic inequality has received additional support from the hard work (both research and its effective dissemination) of Pickett and Wilkinson and colleagues, through their non-profit Equality Trust (2025).
- The PHP’s strong conviction, that socio-economic gradients in health are key to understanding and measuring how well any society is taking care of its citizens’ health, has been vindicated in the subsequent work of many scholars, especially Sir Michael Marmot and colleagues who led the influential WHO Commission on the Social Determinants of Health, completed in 2008 (Marmot et al., 2008). On the other hand, most OECD countries still do not regularly monitor such health inequalities (Frank et al., 2020), and research studies over recent decades around the globe do not show significant reductions in such gradients, even when governments appear to have believed strongly that they should and can be reduced through evidence-based policy action (Mackenbach, 2012).
- Strong policy relevance has been clearly demonstrated for the Program’s work emphasizing the key role of early childhood development in optimizing the health, wellbeing and productivity of entire societies. This work, begun by the PHP in the 1990 s, was subsequently led by another CIAR program – in Human Development (with some overlap in membership, including Clyde Hertzman). Several countries have used CIAR-championed tools, such as the EDI, to improve the equity and overall levels of student education attainment – notably Australia. But much remains to be done, if that approach is to inform early childhood education policies and programs globally.
- Perhaps the most positive spin-off from the CIAR PHP’s work is the current mushrooming of academic and policy literature – especially in Canada – about how to implement and robustly evaluate Population Health Interventions capable of improving societal health overall, as well as reducing health disparities.
- At least one of the original PHP members has published a reflective review of how some key elements of the conceptual framework of the PHP probably need to be revisited and revised in light of the many changes, and emerging challenges, in the field over the last few decades (Frank et al., 2020). Interestingly, his co-authors on this paper include some of the most prominent names in the field of Health Promotion, which was itself rather at odds with the PHP in the 1990 s; it would appear that “that hatchet has now been buried.”

To summarize, there is much to celebrate, yet much left unfinished, a quarter-century after the CIAR PHP was in its heyday. For those accomplishments, based on the Program’s groundbreaking ideas, it is important to give thanks and be proud of what that quintessentially Canadian group of scholars did: their legacy lives on.

### This article is dedicated to the respectful memory of Fraser Mustard and Clyde Hertzman – may they rest in peace

**Contributions to knowledge**

- This summary of the original key ideas of the CIAR Population Health Program (PHP) is the first published by a Program member, in the two decades since the Program ended, and provides succinct historical documentation of one the major Canadian intellectual contributions to modern thinking about the determinants of human health globally.
- Because of the large volume of PHP members’ writings, and the multidisciplinary diversity of the group, this brief summary may be particularly helpful to those who are rather daunted by the original PHP publications, since it classifies the PHP’s major tenets about the determinants of health into four categories: The (Limited) Role of Medical Care; the Influence of Societal/Cultural Characteristics; the Social Micro-Environment around the Person/Individual Socio-Economic Status; the Influence of Daily Stressors and Supports over the Life-Course (mediated by psycho-neuro-endocrine/immunological pathways).
- The many references provide an update on not only how these basic tenets have fared in the global research literature in the last two decades, but also examples of how they have, or have not, led to policy, program and practice innovations, across Canada and in Scotland.

## Data Availability

No data or material were used in writing this paper, apart from the cited references.
